# Effects of ibrutinib on proliferation and histamine release in canine neoplastic mast cells

**DOI:** 10.1111/vco.12520

**Published:** 2019-08-13

**Authors:** Susanne Gamperl, Gabriele Stefanzl, Barbara Peter, Dubravka Smiljkovic, Karin Bauer, Michael Willmann, Peter Valent, Emir Hadzijusufovic

**Affiliations:** ^1^ Department of Internal Medicine I, Division of Hematology & Hemostaseology Medical University of Vienna Vienna Austria; ^2^ Ludwig Boltzmann Institute for Hematology and Oncology Medical University of Vienna Vienna Austria; ^3^ Department/Hospital for Companion Animals and Horses University Clinic for Small Animals, Internal Medicine Small Animals, University of Veterinary Medicine Vienna Austria

**Keywords:** BTK, canine neoplastic mast cells, histamine release, ibrutinib, IgE

## Abstract

The Bruton's tyrosine kinase (BTK) inhibitor ibrutinib is effective in the treatment of human chronic lymphocytic leukaemia and mantle cell lymphoma. Recent data have shown that ibrutinib also blocks IgE‐dependent activation and histamine release in human basophils (BAs) and mast cells (MCs). The aim of this study was to investigate whether BTK serves as a novel therapeutic target in canine mast cell tumours (MCTs). We evaluated the effects of ibrutinib on two canine MC lines, C2 and NI‐1 and on primary MCs obtained from canine MCTs (n = 3). Using flow cytometry, we found that ibrutinib suppresses phosphorylation of BTK and of downstream STAT5 in both MC lines. In addition, ibrutinib decreased proliferation of neoplastic MCs, with IC_50_ values ranging between 0.1 and 1 μM in primary MCT cells and between 1 and 3 μM in C2 and NI‐1 cells. In C2 cells, the combination “ibrutinib + midostaurin” produced synergistic growth‐inhibitory effects. At higher concentrations, ibrutinib also induced apoptosis in both MC lines. Finally, ibrutinib was found to suppress IgE‐dependent histamine release in primary MCT cells, with IC_50_ values ranging from 0.05 to 0.1 μM in NI‐1 cells, and from 0.05 to 1 μM in primary MCT cells. In summary, ibrutinib exerts anti‐proliferative effects in canine neoplastic MCs and counteracts IgE‐dependent histamine release in these cells. Based on our data, ibrutinib may be considered as a novel therapeutic agent for the treatment of canine MCT. The value of BTK inhibition in canine MCT patients remains to be elucidated in clinical trials.

## 1 INTRODUCTION

Mast cell tumours (MCTs) are one of the most frequently diagnosed skin neoplasms in dogs.^1^, ^2^, ^3^, ^4^ These tumours are a heterogeneous group of neoplasms.^2^, ^3^, ^4^ Clinical presentations range from low‐grade, benign tumours with favourable outcome to high‐grade, aggressive disease variants with rapid proliferation of neoplastic MCs leading to a poor prognosis.^4^, ^5^, ^6^ Additional clinical symptoms, such as pruritus or oedema, can be provoked by MC‐derived mediators, such as histamine.^4^, ^7^ These mediator‐induced effects can often be controlled by histamine receptor (HR)1 and HR2 antagonists.^3^, ^8^, ^9^ At present, the only curative treatment approach is wide‐margin surgery.^5^ If a complete resection is not possible or if a tumour recurs, radio‐ and/or chemotherapy are usually offered.^5^, ^10^, ^11^, ^12^, ^13^, ^14^ Recently, masitinib and toceranib, two targeted drugs directed against proto‐oncogene, receptor tyrosine kinase (KIT), have been approved for therapy of non‐resectable or metastasized high‐grade MCT.^15^, ^16^ These drugs have been shown to be effective in several MCT patients.^15^, ^16^, ^17^ Therefore, current efforts focus on the identification and preclinical development of novel targeted drugs that can counteract growth and activation of canine MCT cells.

In humans, the Bruton's tyrosine kinase (BTK) inhibitor ibrutinib is a promising drug that has recently received the Food and Drug Administration approval for the treatment of chronic lymphocytic leukaemia and mantle cell lymphoma.^18^, ^19^, ^20^ In addition, BTK has been reported to be a critical signalling molecule mediating growth and survival of various tumour cells, including neuroblastoma cells and neoplastic human MCs.^21^, ^22^ Furthermore, BTK has been described to play an important role as a target in the downstream pathway of IgE receptor‐cross‐linked human basophils (BAs) and MCs.^23^, ^24^, ^25^, ^26^ More recently, studies published by our and other groups have identified ibrutinib as a potent blocker of IgE‐dependent activation and histamine release in human BAs.^26^, ^27^ However, only few reports exist about the *in vitro* and *in vivo* effects of ibrutinib in dogs. Recently, it has been described that ibrutinib exerts anti‐tumour effects in naturally occurring B cell non‐Hodgkin‐lymphoma in canine patients.^28^ Based on these data, ibrutinib may be an interesting agent to test in comparative oncology contexts. We were interested to examine the effect of ibrutinib on canine neoplastic MCs. The specific aims of our study were to examine whether ibrutinib may serve as a potential new drug for treatment of canine MCT and whether ibrutinib is able to suppress histamine release in neoplastic MCs.

## 2 MATERIALS AND METHODS

### 2.1 Drugs and reagents

Ibrutinib was obtained from Selleck Chemicals (Houston, Texas), toceranib from Sigma‐Aldrich (St. Louis, Missouri), masitinib and midostaurin from LC laboratories (Woburn, Massachusetts). Stock solutions for all drugs were prepared by dissolving in dimethyl sulfoxide (DMSO) purchased from Sigma‐Aldrich. RPMI 1640 medium, Iscove's modified Dulbecco's medium (IMDM) and antibiotics (penicillin, streptomycin) were purchased from Lonza (Basel, Switzerland), amphotericin B from PAN‐Biotech (Aidenbach, Germany), fetal calf serum (FCS) from Gibco Life Technologies (Carlsbad, California), ^3^H‐thymidine from PerkinElmer (Waltham, Massachusetts), collagenase type 2 from Worthington (Lakewood, New Jersey) and trypan blue and 4′,6‐diamidino‐2‐phenylindole (DAPI) from Sigma‐Aldrich. DMSO was used as vehicle‐control in all experiments (corresponding to highest drug concentrations) and showed no effects on growth and activation of canine MCs (not shown).

### 2.2 Cell lines and culture conditions

Two canine mastocytoma cell lines were used: C2 and NI‐1. C2 cells were kindly provided by Dr. Warren Gold (Cardiovascular Research Institute, University of California, San Francisco, California).^29^ NI‐1 cells were established in our laboratory as described previously.^30^ Both cell lines were cultured in RPMI 1640 medium containing 10% FCS, antibiotics and amphotericin B. The human MC line HMC‐1 was kindly provided by Dr. Joseph H. Butterfield (Mayo Clinic, Rochester, Minnesota) and cultured in IMDM plus 10% FCS, alpha‐thioglycerol, antibiotics and amphotericin B.^31^ Cell lines were kept in culture at 5% CO_2_ and 37°C for 6 to 8 weeks. Thereafter, cells were discarded and new cells were thawed from an original stock.

### 2.3 Isolation of primary canine neoplastic MCs from mastocytoma specimens

Fresh MCT samples were obtained from three dogs undergoing surgery at the University of Veterinary Medicine Vienna (Vienna, Austria). Detailed characteristics of mastocytoma patients are listed in Table 1. Primary neoplastic MCs were isolated using collagenase as previously published.^32^ In brief, tissue samples were cut into small pieces, washed thoroughly in Tyrode's buffer and were then incubated in 75 mg collagenase type 2 dissolved in 50 mL 0.9% NaCl at 37°C for 180 minutes. Isolated MCs were recovered by filtration through cell strainer (70 μM pore size) and collected in FBS‐containing tubes. After washing, cells were examined for viability (trypan blue exclusion) and MC numbers (Wright Giemsa staining).

**Table 1:** Canine mastocytoma patients' characteristics

No.	Sex (f/m)	Age (years)	Breed	Grade^a^	Isolated MCT cells were used for
1	f	12	Wire‐haired Dachshund	II	Histamine release
2	m	7.8	Chihuahua	III	^3^H‐thymidine uptake, histamine release
3	mc	9	American Staffordshire Terrier	III	^3^H‐thymidine uptake

Abbreviations: f, female; m, male; mc, male castrated; MCT, mast cell tumour.

a Grade according to the Patnaik et al scheme.^58^

### 2.4 Western blot analysis of BTK and STAT5 expression in MC lines

Western blot experiments were performed using C2 and NI‐1 cells essentially as reported.^33^ A detailed description of the technique is provided in the Method Section of the Supplement.

### 2.5 Analysis of BTK‐ and STAT5 phosphorylation by flow cytometry

For staining of phosphorylated (p) BTK and pSTAT5, C2 cells and NI‐1 cells were incubated in control medium or in medium containing increasing concentrations of ibrutinib (0.1‐10 μM) at 37°C for 4 hours. Then, cells were permeabilized by methanol (−20°C, 15 minutes) and incubated with Alexa Fluor 647‐labelled mouse monoclonal antibody (mAb) N35‐86 against pBTK (phosphorylation site: Y223) or Alexa Fluor 647‐labelled mouse mAb 47 against pSTAT5 (phosphorylation site: Y694) (BD Biosciences, San José, California) for 30 minutes. A mouse mAb MOPC‐21 against IgG1 (BD Biosciences) was used as isotype control. Expression of pBTK and pSTAT5 was quantified by multicolor flow cytometry on a FACSCanto (BD Biosciences).

### 2.6 Measurement of proliferation of canine neoplastic MCs

MC lines and primary mastocytoma cells were seeded in flat‐bottom 96‐well microtiter plates (5 × 10^3^ cells/well) and incubated in control medium, vehicle control or various concentrations of ibrutinib (0.01‐5 μM) at 37°C for 48 hours. To evaluate potential cooperative drug effects, cells were exposed to ibrutinib, midostaurin, toceranib, masitinib (as single agent) or to drug combinations, namely “ibrutinib + midostaurin”, “ibrutinib + toceranib”, and “ibrutinib + masitinib,” at a fixed ratio of drug concentrations. After incubation, 0.5 μCi of ^3^H‐thymidine was added (37°C, 16 hours). Thereafter, cells were harvested on filter membranes (Packard Bioscience, Meriden, Connecticut) in a Filtermate 196 harvester (Packard Bioscience). Filters were air‐dried and the bound radioactivity was measured in a β‐counter (TopCount NXT, Packard Bioscience). All experiments were performed in triplicates.

### 2.7 Evaluation of apoptosis in drug‐exposed cells

C2 and NI‐1 cells were incubated in control medium, vehicle control or in various concentrations of ibrutinib (1‐25 μM) at 37°C for 24 or 48 hours. After incubation, the percentages of viable, apoptotic and necrotic cells were quantified by microscopy on slides stained with the differential Quick Stain Kit (Modified Wright Giemsa, Sigma‐Aldrich). In addition, apoptosis was assessed by Annexin V (eBioscience, San Diego, California) and DAPI staining by flow cytometry as described.^31^ In brief, cells were incubated in control medium or medium containing various concentrations of ibrutinib (1‐25 μM) at 37°C for 24 and 48 hours. Thereafter, cells were harvested and incubated with Annexin V in binding‐buffer. Then, DAPI (1 μg/mL) was added and cells were analysed by flow cytometry on a FACSCanto (BD Biosciences). The total fraction of all apoptotic cells was quantified by determining the percentage of Annexin V‐single positive (early apoptotic) cells plus the percentage of Annexin V plus DAPI positive (late apoptotic) cells relative to all cells captured in the sample tube.

### 2.8 Histamine release experiments

NI‐1 cells and primary mastocytoma cells were sensitized by preloading with 5 μg/mL purified dog IgE (Bethyl, Montgomery, Alabama) at 37°C for 2 hours. Cells without IgE preloading were used as negative control. Thereafter, the cells were incubated with different concentrations of ibrutinib (0.001‐1 μM) at 37°C for 60 minutes. After incubation, cells were washed in phosphate‐buffered saline (PBS, Gibco Life Technologies), resuspended in histamine release buffer (HRB, Immunotech, Marseille, France) and then incubated with 5 μg/mL goat anti‐dog‐IgE antibody (Bethyl Laboratories) at 37°C for 30 minutes. After incubation, cells were centrifuged at 4°C, and the cell‐free supernatants and cell pellets were recovered and analysed for histamine content by radioimmunoassay (RIA, Immunotech). Total histamine was measured in lysates of non‐sensitized cells and served as reference for calculating the percentage of released histamine of sensitized and drug‐exposed cells. All experiments were performed in triplicates.

### 2.9 Statistical analysis

Data were presented as mean values from at least three independent experiments with standard deviation (SD). To determine the significance in differences seen between drug‐exposed and untreated cells, the Student's *t* test for independent samples was applied. Results were considered statistically significant when *P* was <0.05. Effects of drug combinations were determined using Calcusyn software (Biosoft, Ferguson, Missouri) and expressed as combination index (CI) values. Drug effects were considered to be synergistic when the CI was <1.

## 3 RESULTS

### 3.1 Effects of ibrutinib on pBTK and pSTAT5 expression in canine neoplastic MCs

**Figure 1:**
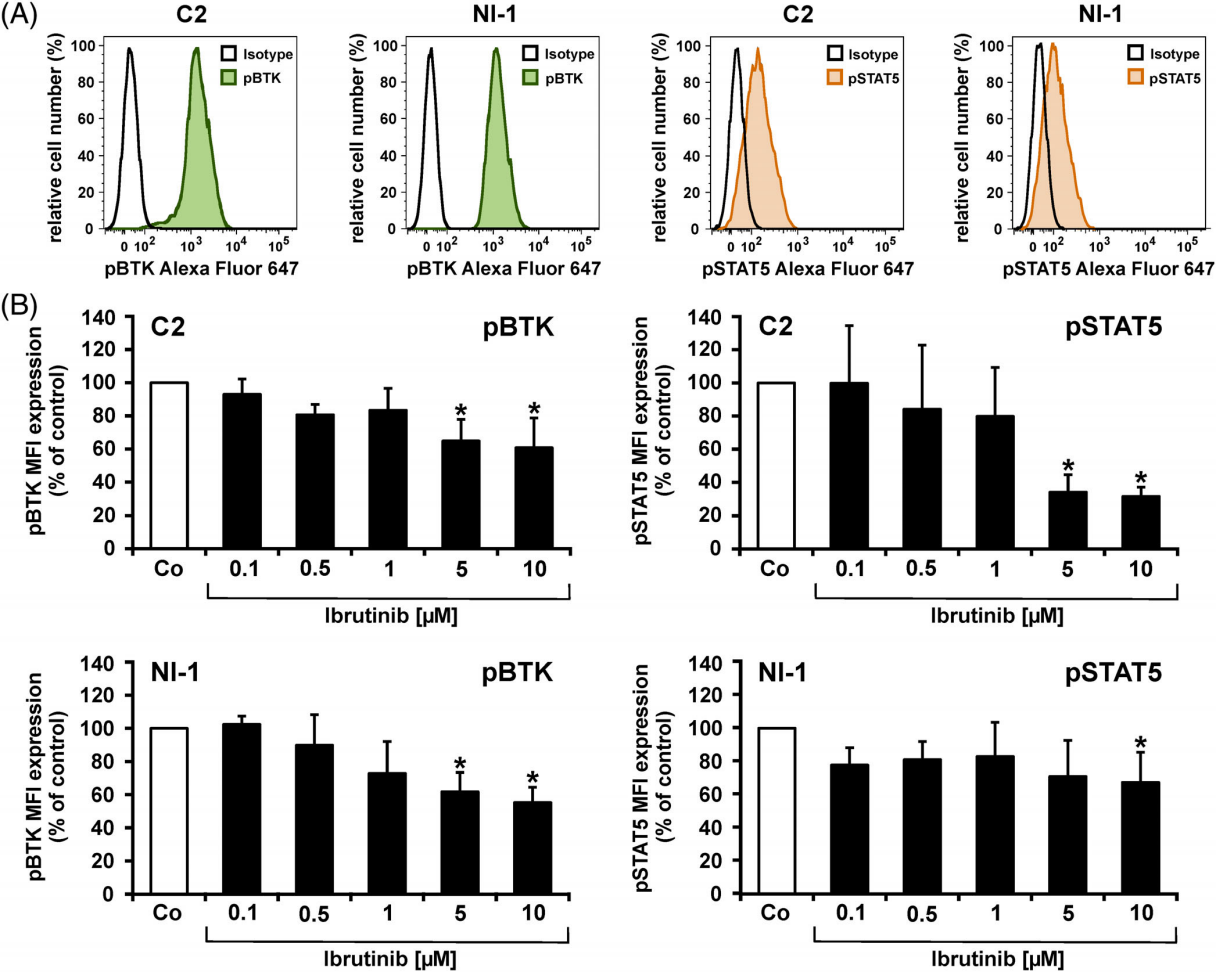
Detection of phosphorylated (p) Bruton's tyrosine kinase (BTK) and pSTAT5 in canine mast cell (MC) lines. A, C2 and NI‐1 cells were permeabilized by methanol and incubated with a monoclonal antibody against pBTK and pSTAT5 for 30 minutes. After incubation, cells were washed, and expression of pBTK and pSTAT5 was determined by flow cytometry. Expression levels of pBTK (green histograms) and pSTAT5 (orange histograms) are shown in comparison to the isotype control (black histograms). B, C2 and NI‐1 cells were incubated in control medium (Co) or in increasing concentrations of ibrutinib (0.1‐10 μM) at 37°C for 4 hours. Then, cells were analysed for pBTK (left panels) or pSTAT5 (right panels) expression as described in (A). Results show median fluorescence intensity (MFI) values expressed as % of control and represent the mean ± SD from four independent experiments. *P < 0.05 compared with control (Co) [Colour figure can be viewed at http://wileyonlinelibrary.com]

To test the effects of ibrutinib on BTK activation and downstream STAT5 activation, we examined the phosphorylation status of BTK and STAT5 using flow cytometry in drug‐exposed cells. We found that C2 cells and NI‐1 cells constitutively express pBTK and pSTAT5 (Figure 1A). Exposure of C2 cells and NI‐1 cells to various concentrations of ibrutinib resulted in a concentration‐dependent decrease in expression of pBTK and pSTAT5 (Figure 1B). In time course experiments, the inhibitory effects of ibrutinib on expression of pBTK and pSTAT5 were seen at all time points examined (0.25‐4 hours) (Figure S1). As assessed by Western blotting, both canine MC lines (C2 and NI‐1) expressed the BTK and STAT5 protein (Figure S2). The human MC line HMC‐1.2 served as positive control in these experiments (Figure S2).

### 3.2 Effects of ibrutinib on proliferation of canine neoplastic MCs

**Figure 2:**
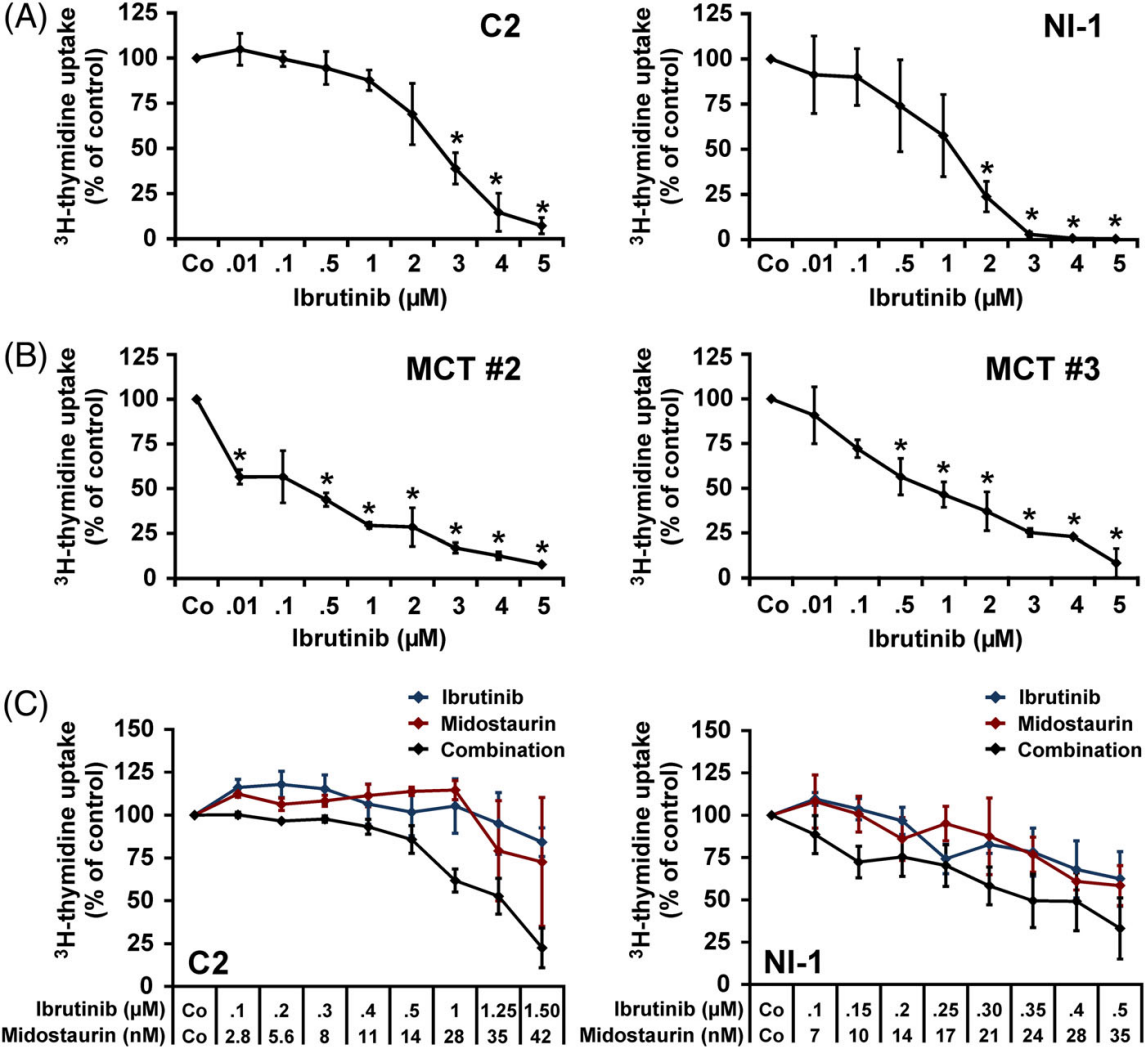
Effects of ibrutinib or “ibrutinib + midostaurin” on proliferation in canine neoplastic mast cells (MCs). C2 and NI‐1 cells (A) or primary cells isolated from mastocytoma patients #2 and #3 (B) were incubated in control medium (Co) or in various concentrations of ibrutinib (0.01‐5 μM) at 37°C for 48 hours. C, For assessing drug combination effects, C2 and NI‐1 cells were incubated in Co, ibrutinib (blue line), midostaurin (red line) or in a combination of both drugs (black line) at a fixed ratio of drug concentrations (as indicated) at 37°C for 48 hours. After incubation, ^3^H‐thymidine was measured. Results show ^3^H‐thymidine uptake as percentage of control (=100%, Co) and represent the mean ± SD of four independent experiments (C2, NI‐1) or the mean ± SD of triplicates (primary MCs). *P < 0.05 compared with control (Co) [Colour figure can be viewed at http://wileyonlinelibrary.com]

As determined by ^3^H‐thymidine uptake, ibrutinib was found to decrease proliferation of C2 cells and NI‐1 cells with IC_50_ values ranging between 1 and 3 μM (Figure 2A, Table 2). Ibrutinib also suppressed proliferation in primary MCT cells with IC_50_ values ranging between 0.1 and 1 μM (Figure 2B, Table 2). Synergistic effects between ibrutinib and midostaurin on proliferation were seen in C2 cells, but not in NI‐1 cells (Figure 2C). The drug combinations “ibrutinib + masitinib” and “ibrutinib + toceranib” induced some cooperative growth‐inhibitory effects in C2 and NI‐1 cells, although synergistic interactions were not seen (Figure S3).

**Table 2:** Effects of ibrutinib on proliferation of canine neoplastic mast cells

Cells	IC_50_ values^a^ obtained with ibrutinib (μM)
C2	2‐3
NI‐1	1‐2
MCT #1	n.d.
MCT #2	0.1‐0.5
MCT #3	0.5‐1

Abbreviations: IC_50_, half maximal inhibitory concentration; n.d., not determined; μM, micromolar.

a Assessed by ^3^H‐thymidine uptake.

### 3.3 Effects of ibrutinib on survival of C2 cells and NI‐1 cells

**Figure 3:**
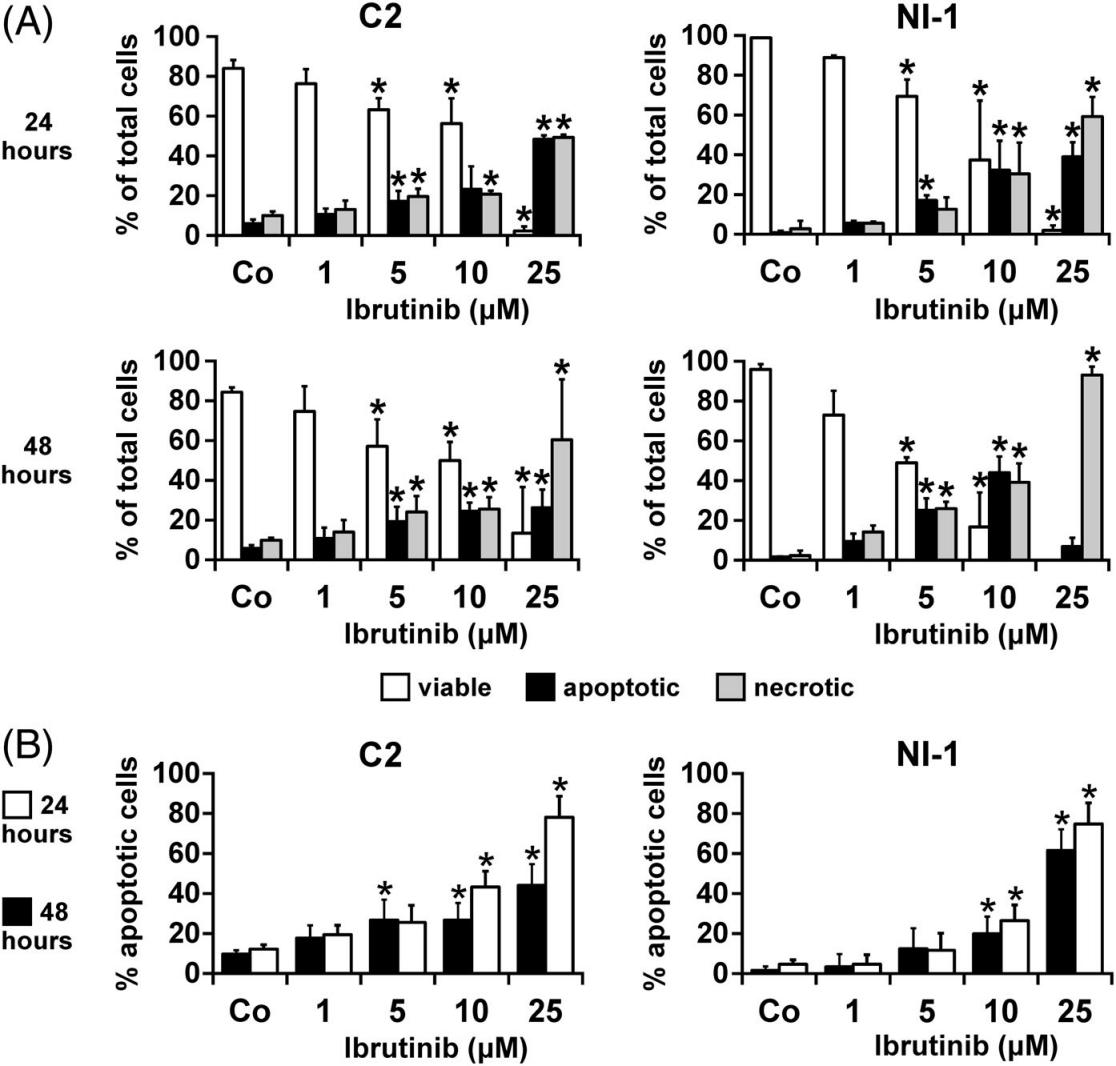
Effects of ibrutinib on survival of canine mast cell (MC) lines. A, C2 cells (left panels) and NI‐1 cells (right panels) were incubated in control medium (Co) or in various concentrations of ibrutinib (1‐25 μM) at 37°C for 24 or 48 hours (as indicated). After incubation, the percentages of viable (open columns), apoptotic (black columns) and necrotic (grey columns) cells were quantified by light microscopy on Giemsa‐stained slides and the percentage of total cells was calculated. B, C2 cells (left panel) and NI‐1 cells (right panel) were incubated as described in (A). After incubation, cells were examined by Annexin V‐ and DAPI staining (flow cytometry). Results show the percentage of all apoptotic cells, determined by adding the percentage of cells stained only positive for Annexin V (early apoptosis) plus the percentage of cells “double‐positive” for Annexin V and DAPI (late apoptosis). Results represent the mean ± SD of three independent experiments in each cell line. *P < 0.05 compared with control (Co)

In a next step, we examined whether ibrutinib induces apoptosis in canine MC lines. As assessed by microscopy, ibrutinib induced apoptosis and consecutive necrosis in both MC lines with EC_50_ values ranging between 10 and 25 μM for C2 cells and between 5 and 10 μM for NI‐1 cells after 48 hours (Figure 3A). Apoptosis induction was confirmed in Annexin V/DAPI staining experiments, where ibrutinib decreased survival with EC_50_ values ranging from 10 to 25 μM after 48 hours (Figure 3B).

### 3.4 Effects of ibrutinib on IgE‐dependent histamine release in NI‐1 cells and primary mastocytoma cells

**Figure 4:**
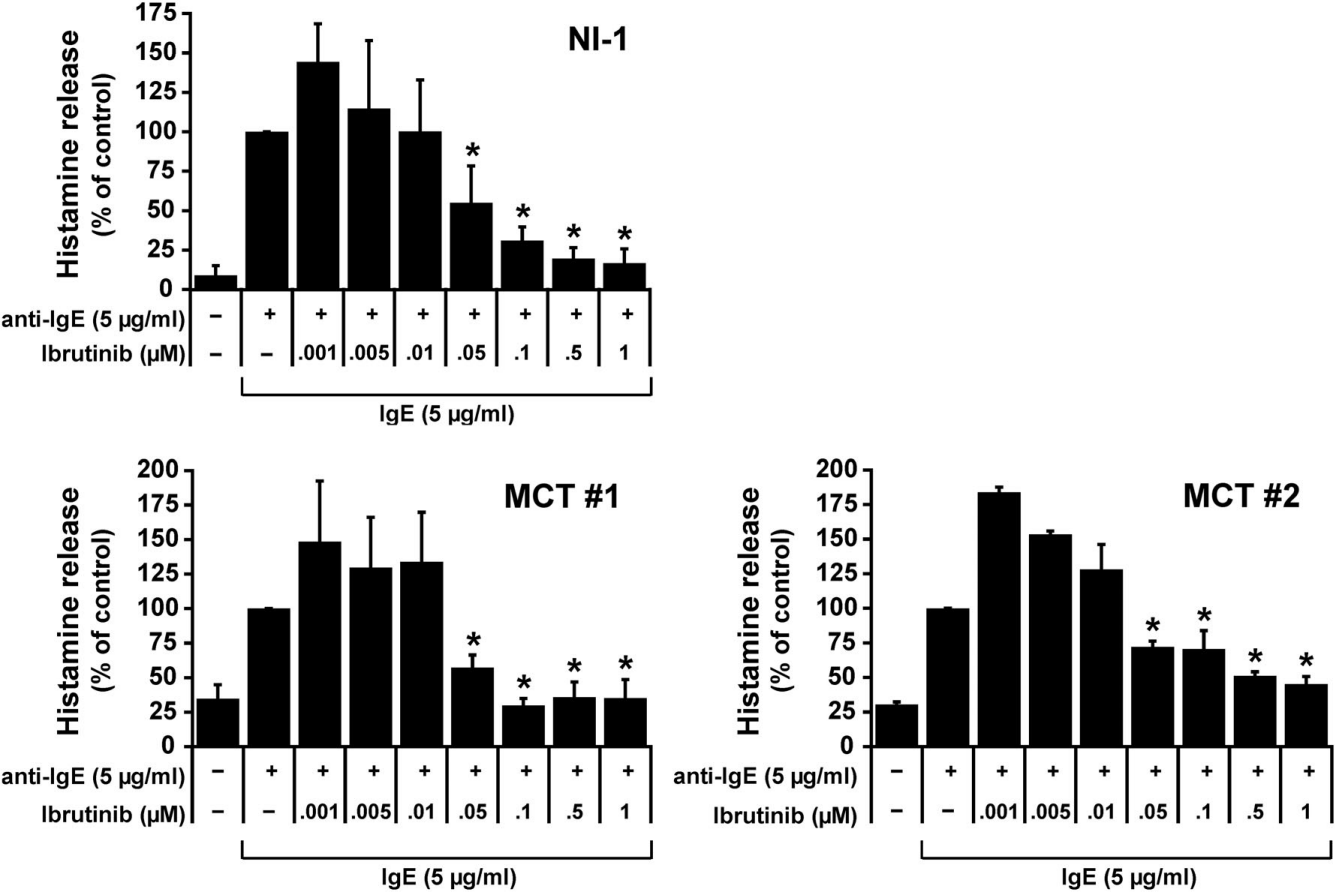
Effects of ibrutinib on IgE‐dependent histamine release in canine neoplastic mast cells (MCs). NI‐1 cells (upper panel) and primary cells isolated from mastocytoma patients #1 and #2 (lower panels) were incubated in medium alone or in medium containing 5 μg/mL IgE at 37°C for 2 hours. Then, cells were incubated in medium without ibrutinib or in medium containing increasing concentrations of ibrutinib (0.001‐1 μM) at 37°C for 60 minutes. Thereafter, histamine release was induced by adding 5 μg/mL anti‐IgE at 37°C for 30 minutes. Histamine release was calculated as percentage of total (cellular) histamine. Drug effects were expressed as relative decrease of the percent histamine release induced by “IgE + anti‐IgE” (=100%). Results represent the mean ± SD of at least three independent experiments. *P < 0.05 compared with the control (=“IgE + anti‐IgE”)

Ibrutinib has recently been shown to counteract IgE‐dependent activation of human BAs.^26^, ^27^ To examine whether ibrutinib exerts inhibitory effects on IgE‐dependent histamine release in canine neoplastic MCs, we performed experiments using NI‐1 cells, known to express a functional IgE receptor^30^, ^34^ and primary mastocytoma cells. In both cell types, ibrutinib was found to inhibit IgE‐dependent histamine release in a concentration‐dependent manner with IC_50_ values ranging between 0.05 and 0.1 μM in NI‐1 cells and between 0.05 and 1.0 μM in primary MCT cells (Figure 4).

## 4 DISCUSSION

Aggressive canine MCT is a disease with an unmet therapeutic need.^2^, ^3^, ^4^, ^5^ In human and canine MC neoplasms, mutated KIT can induce growth‐factor independent activation and uncontrolled proliferation of neoplastic MCs.^2^, ^6^, ^35^, ^36^, ^37^, ^38^ The tyrosine kinase inhibitors (TKIs) masitinib and toceranib are already in use to treat advanced MC neoplasms in dogs.^15^, ^16^ However, although these drugs show clinical efficacy in a subset of canine MCT patients, relapses are frequently seen.^17^, ^39^ Therefore, identification of new molecular targets and development of novel drugs are of great importance. We here describe that the BTK‐targeting drug ibrutinib is a potent inhibitor of growth of canine neoplastic MCs. In addition, we show that ibrutinib suppresses IgE‐dependent histamine release in neoplastic canine MCs. These observations may be relevant clinically and may lead to the development of new treatment strategies in dogs suffering from MCT.

As BTK is the major target of ibrutinib,^28^, ^40^ we were first interested to demonstrate the expression of the BTK protein in both MC lines. In addition, we were able to show that both cell lines exhibit phosphorylated BTK and the downstream effector molecule STAT5. This observation confirms previous reports on STAT5/pSTAT5 expression in canine MC lines.^41^ Furthermore, we found that ibrutinib rapidly suppresses phosphorylation of both signalling molecules in a concentration‐dependent manner in these cells. Downregulation of pBTK by ibrutinib is consistent with data demonstrated in B‐cell neoplasms, such as chronic lymphocytic leukaemia.^19^, ^42^

BTK has also been described to be a downstream molecule in the KIT‐signalling pathway, known to be critical for MC growth, proliferation and survival.^43^ We were able to show that ibrutinib counteracts proliferation of C2 and NI‐1 cells with IC_50_ values between 1 and 3 μM, and of primary MCT cells with IC_50_ values between 0.1 and 1 μM. The IC_50_ values obtained with primary MCT cells were lower when compared with MC lines. One possible explanation for this phenomenon may be that both MC lines represent an aggressive type of disease. Both MC lines originate from cells selected for high doubling times by continuous *in vitro* culturing, and therefore, they derive from the fastest growing subclone of the original tumour. In addition, C2 cells were generated by serial passaging through a mouse model and NI‐1 cells were established from a dog with MC leukaemia.^29^, ^30^ An additional factor contributing to higher sensitivity of primary MCT cells may be their dependence on the microenvironment that was lost for C2 and NI‐1 cells.^44^ We screened the literature for tolerable ibrutinib doses and found that human patients suffering from chronic lymphocytic leukaemia tolerate daily administration of 560 mg ibrutinib corresponding to a steady state plasma level of 2.16 μM (953 ng/mL).^45^ Based on this information, the calculated tolerable dose of ibrutinib in an average male person (body weight: 80 kg) would be 7 mg/kg per day. Honigberg et al reported that dogs tolerate even higher doses of 20 mg/kg per day for up to 35 days without relevant side effects.^28^ However, plasma levels that can be reached in dogs after administration of 20 mg/kg of ibrutinib per day have not been reported.

As growth‐inhibitory effects of TKIs on neoplastic cells are often associated with disrupted tyrosine kinase activity and consecutive apoptosis and as BTK has been shown to be important for the survival of mantle lymphoma cells,^46^ we investigated the effects of ibrutinib on viability of C2 and NI‐1 cells. Ibrutinib was found to induce apoptosis only when applied at higher concentrations than in our growth‐inhibition experiments. EC_50_ values obtained with ibrutinib ranged between 10 and 25 μM for both MC lines, which is consistent to findings in mantle lymphoma cell lines, Mino and JeKo‐1, where EC_50_ values ranged between 1.0 and 10 μM and between 10 and 25 μM, respectively.^46^ Moreover, recent data have shown that ibrutinib does not induce apoptosis in lymphoid malignancies in pharmacologically achievable concentrations.^47^, ^48^, ^49^

Neoplastic MCs harbouring *KIT*‐mutations may gain resistance against TKIs; therefore, current research in canine and human MC neoplasms focuses on drug combinations to overcome this problem.^33^, ^50^ In the current study, we were interested to examine cooperative effects between ibrutinib and KIT‐targeting drugs. Midostaurin is a KIT‐targeting drug that is well known to suppress the growth of human KIT D816V+ MCs *in vitro* and *in vivo*.^50^, ^51^, ^52^ We found that the drug combination “ibrutinib + midostaurin” exerts synergistic growth‐inhibitory effects in C2 cells (CI < 1), but not in NI‐1 cells. A possible explanation for the weaker effect of the drug combination “ibrutinib + midostaurin” in NI‐1 cells may be the presence of several different mutations in *KIT*. Notably, whereas C2 cells contain only one *KIT* mutation, NI‐1 cells are known to display six different *KIT* mutations, which may result in a faster proliferation of NI‐1 cells and possibly also in a more resistant phenotype.^30^ We also tested the effects of other drug combinations in C2 and NI‐1 cells. Toceranib and masitinib are two KIT‐targeting drugs that are used to treat canine MCT patients in daily practice. We found that the drug combinations “ibrutinib + toceranib” and “ibrutinib + masitinib” exert cooperative growth‐inhibitory effects in both MC lines. However, synergistic growth‐inhibitory effects were not seen.

In patients suffering from MC neoplasms, clinical symptoms may also arise from effects of MC‐derived mediators.^4^, ^7^ In the current study, we tested the effects of ibrutinib on IgE‐dependent histamine release in NI‐1 cells and primary MCT cells. C2 cells could not be employed in these experiments because they do not express a functionally active IgE receptor.^53^ The IC_50_ values obtained with ibrutinib in NI‐1 cells as well as in primary mastocytoma cells were found to be within a “pharmacological” range (0.05‐1 μM). Since concentrations required for blocking histamine release were significantly lower than for blocking proliferation and survival, ibrutinib may also have clinical implications for other IgE‐dependent diseases in dogs, such as canine atopic dermatitis or food allergies.^54^, ^55^, ^56^, ^57^ Our findings are reinforced by previous data showing that BTK is expressed in human BAs and MCs and that BTK inhibition by ibrutinib is associated with reduced IgE‐dependent mediator release in these cells.^23^, ^24^, ^25^, ^26^, ^27^

In summary, our data show that ibrutinib exerts anti‐proliferative and survival‐reducing effects in canine neoplastic MCs. Moreover, we found that BTK inhibition by ibrutinib resulted in a concentration‐dependent suppression of IgE‐dependent histamine release in NI‐1 cells and primary MCT cells. These observations suggest that targeting BTK may represent a potential therapeutic approach in canine MCT. The clinical impact of this concept remains to be elucidated.

## CONFLICT OF INTEREST

The authors declare no conflict of interest.

## AUTHOR CONTRIBUTIONS

S.G., P.V. and E.H. designed the study. S.G. performed most experiments, analysed the data and generated figures and tables. G.S. measured histamine release and ^3^H‐thymidine uptake experiments. B.P. performed Western blot experiments. D.S. and K.B. assisted in isolating primary neoplastic MCs from canine tumour samples. D.S., M.W., P.V. and E.H. assisted in the interpretation of data. S.G., M.W., P.V. and E.H. participated in writing and final editing of the manuscript. All authors have read and approved the final manuscript.

## DATA ACCESSIBILITY

Please contact the corresponding authors for all reasonable data requests.

## Supporting information


**Data S1**. Supporting information.
