# Supplementary material for: Effects of ibrutinib on proliferation and histamine release in canine neoplastic mast cells
Source: Vet Comp Oncol. 2019 Aug 13;17(4):553–61. doi: 10.1111/vco.12520 (PMC6900099; doi:10.1111/vco.12520)
Supplement: Supplementary file 1 — Data S1. Supporting information. [file VCO-17-553-s001.docx]

**Supplementary File to Manuscript:**

**Effects of ibrutinib on proliferation and histamine release**

**in canine neoplastic mast cells**

Susanne Gamperl^1^, Gabriele Stefanzl^1,2^, Barbara Peter^1,2^,

Dubravka Smiljkovic^1^, Karin Bauer^2^, Michael Willmann^2,3^,

Peter Valent^1,2^, Emir Hadzijusufovic^1,2,3^

^1^Department of Internal Medicine I, Division of Hematology & Hemostaseology, Medical University of Vienna, Vienna, Austria; ^2^Ludwig Boltzmann Institute for Hematology and Oncology, Medical University of Vienna, Vienna, Austria; ^3^Department/Hospital for Companion Animals and Horses, University Clinic for Small Animals, Internal Medicine Small Animals, University of Veterinary Medicine, Vienna, Austria;

Running Title: Ibrutinib in canine mastocytoma

**Correspondence to:**

Emir Hadzijusufovic, DVM

Ludwig Boltzmann Institute for Hematology and Oncology

Medical University of Vienna, Austria

Waehringer Guertel 18-20,

1090 Vienna, Austria

Phone: +43 1 40400 49990

E-mail: emir.hadzijusufovic@meduniwien.ac.at

This study was supported by the Austrian Science Fund (FWF): DK W1248-B30,

SFB F4701-B20 and SFB F4704-B20

**Supplementary Materials and Methods**

***Western blot analysis of BTK and STAT5 expression in MC lines***

Western blot experiments were performed using C2 and NI-1 cells essentially as reported.^1^ In brief, cell lysates were separated in 7.5% SDS-polyacrylamide gel electrophoresis and transferred to PVDF membranes (GE Healthcare, Buckinghamshire, UK). Membranes were blocked using 5% bovine serum albumin (BSA) for 30 minutes. Thereafter, antibodies directed against BTK (Santa Cruz, Santa Cruz, CA, USA), STAT5 (BD Biosciences, San José, CA, USA) and β-actin (Santa Cruz) were applied according to the manufacturers´ instructions. Antibody reactivity was made visible with donkey anti-rabbit IgG or sheep anti-mouse IgG (both from GE Healthcare) and ECL Plus Western Blotting Substrate (Thermo Scientific, Rockford, IL, USA).

**Supplementary Figures**

**
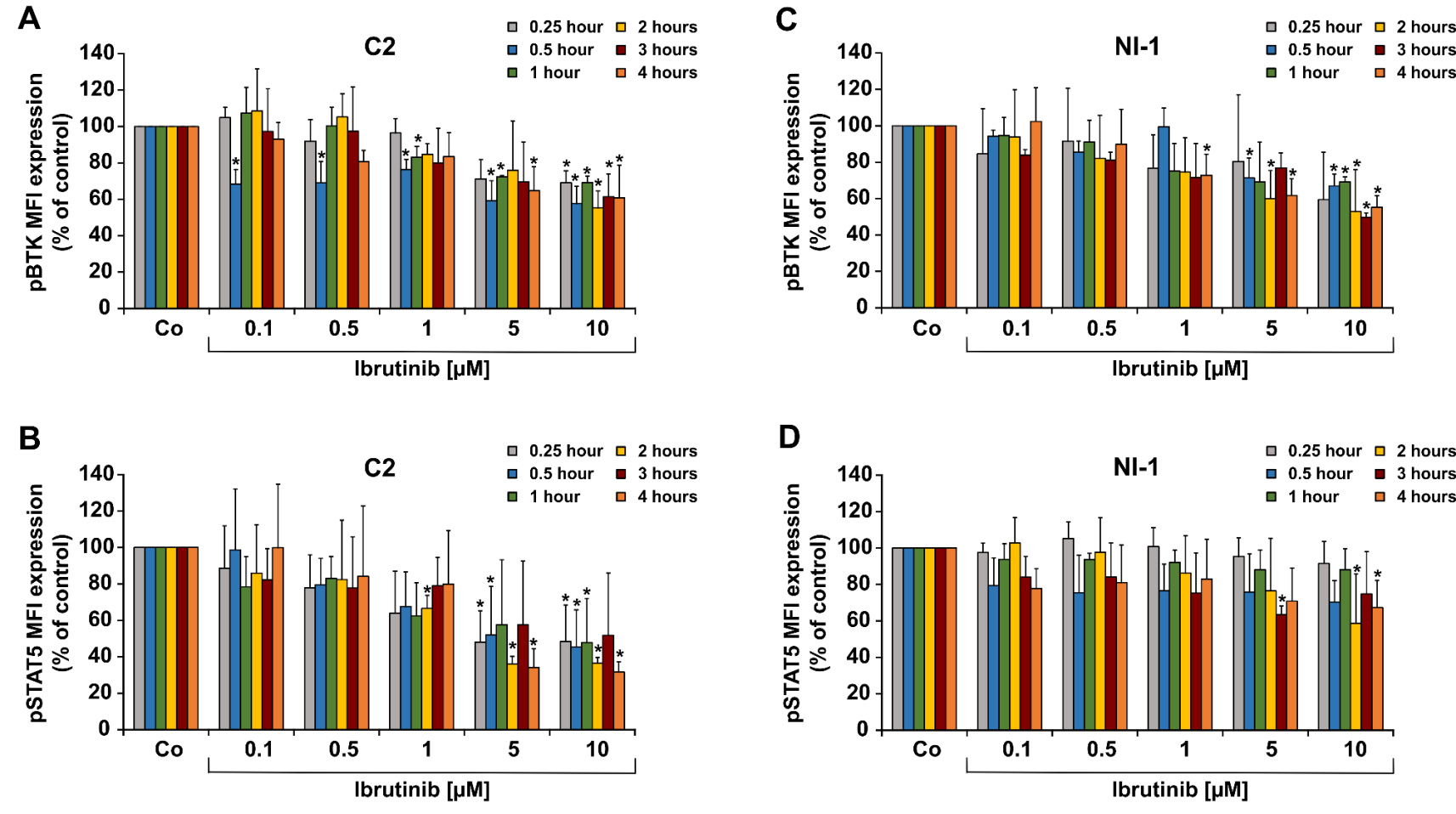
**

***Supplementary Figure S1. Dose- and time-dependent effects of ibrutinib on phosphorylated (p)BTK and pSTAT5 expression in canine MC lines***

C2 cells (A,B) and NI-1 cells (C,D) were incubated in control medium (Co) or in medium containing ibrutinib (0.1-10 µM) at 37°C for 0.25, 0.5, 1, 2, 3 and 4 hours. Thereafter, cells were permeabilized by methanol (-20°C, 15 minutes) and incubated with a monoclonal antibody (mAb) against pBTK (A,C) and pSTAT5 (B,D) for 30 minutes. Then, cells were washed and expression of pBTK and pSTAT5 was determined by flow cytometry. Results show median fluorescence intensity (MFI) values expressed as percentage (%) of control and represent the mean±SD from at least three independent experiments. Asterisk (*): *P*<0.05 compared to control (Co).

***

***

***Supplementary Figure S2. BTK and STAT5 protein expression in canine MC lines***

C2, NI-1 and HMC-1.2 cells were analyzed for expression of total BTK and total STAT5 by Western blotting. β-actin served as loading control. The figure shows one representative out of three Western blot experiments – the 3 experiments showed almost the same results.

**
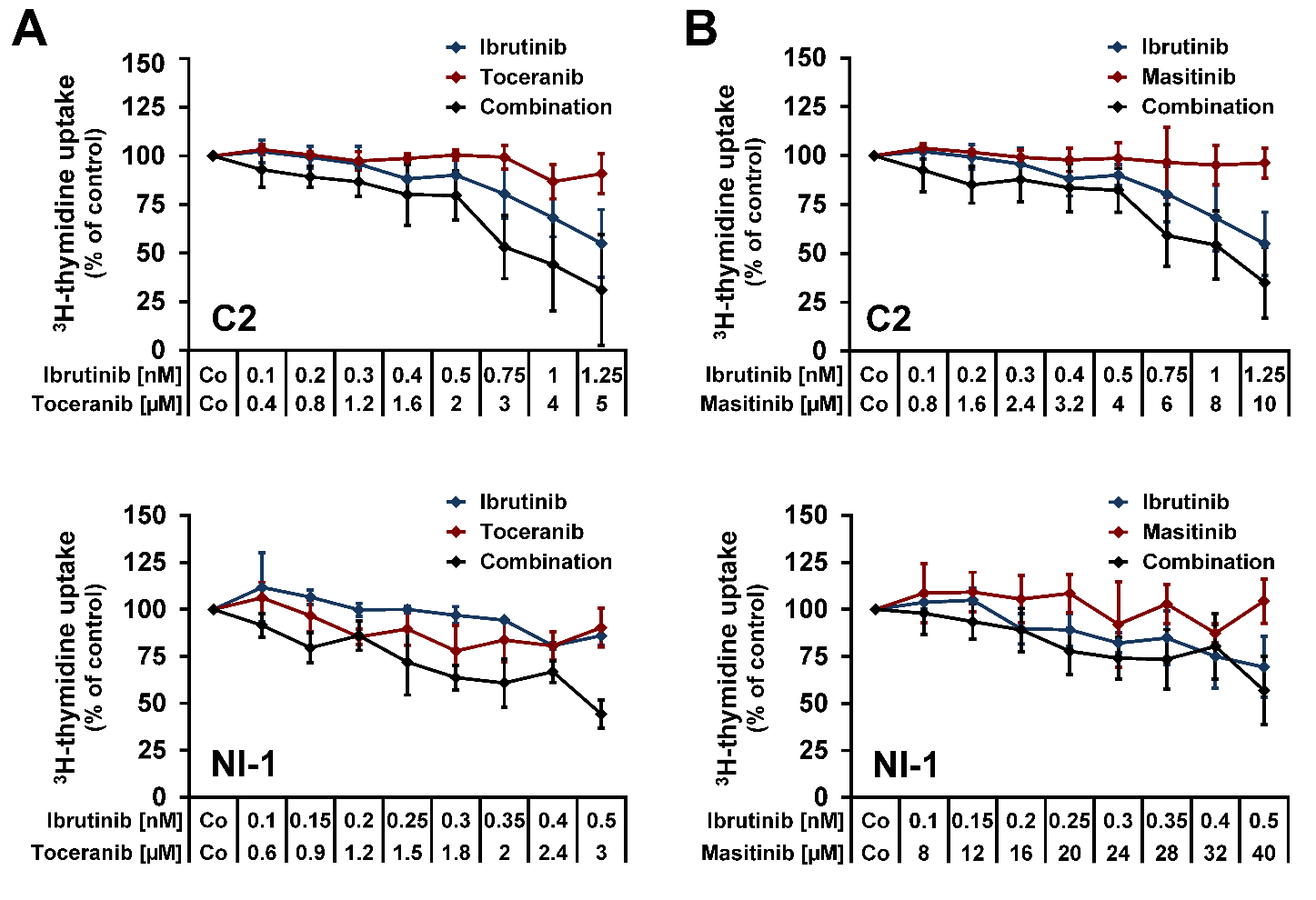
**

***Supplementary Figure S3. Cooperative effects of ibrutinib and toceranib and of ibrutinib and masitinib on proliferation in canine MC lines***

C2 cells and NI-1 cells were incubated in control medium (Co), various concentrations of ibrutinib (blue line), toceranib (A, red line), or masitinib (B, red line) or in a combination of ibrutinib and either toceranib or masitinib (black line) at a fixed ratio of drug concentrations (as indicated) at 37°C for 48 hours. After incubation, ^3^H-thymidine was measured. Results show ^3^H-thymidine uptake as percentage of control (=100%, Co) and represent the mean±SD of three independent experiments in both cell lines.

**References**

1. Gleixner KV, Rebuzzi L, Mayerhofer M, et al. Synergistic antiproliferative effects of KIT tyrosine kinase inhibitors on neoplastic canine mast cells. *Exp Hematol.* 2007;35:1510-1521.
